# Pulpitis in a dens invaginatus presenting as a Trigeminal Neuralgia: A case report

**DOI:** 10.4317/jced.57881

**Published:** 2022-02-01

**Authors:** Salomé Mascarell, Valentin Marchi, Yves Boucher

**Affiliations:** 1 MSc. UFR d’Odontologie, Université de Paris, F-75006, Paris, France; 2 DDS, MSc. Hôpital Bretonneau, APHP Paris, France; 3 DDS, PhD. Laboratoire de Neurobiologie Orofaciale, LabNOF (EA7543), Paris, France & Hôpital Pitié Salpêtrière, APHP, France

## Abstract

**Introduction:**

Orofacial pain diagnosis is a difficult process. This article reports the case of a 38 y.o. patient experiencing severe acute facial pain of dental origin initially diagnosed as non odontogenic.

**Case Report:**

The patient consulted at the dental emergency department for severe and frequent neuropathic-like paroxysmal pain attacks located in the anterior right maxilla. The pain fulfilled the ICHD3 criteria for Trigeminal Neuralgia (TN) of the right maxillary branch (V2) and responses to sensitivity tests were ambiguous due to severe allodynia. After one week of carbamazepine treatment (600mg/day), the patient was pain free except for a slight mechanical allodynia on tooth #12. Sensitivity tests revealed pulp necrosis and tomodensitometry revealed a rare developmental abnormality, dens invaginatus. Root canal treatment was performed.

**Results:**

No recurrence of the pain was noted after 18 months without any medication.

**Conclusions:**

Inflammatory pulpal pain may mimic TN, misleading experienced clinicians.

** Key words:**Dens in dente, neuropathic pain, necrosis, diagnosis, atypical pain, endodontics.

## Introduction

Orofacial pain (OFP) diagnosis is a difficult process. The complex cephalic innervation and the specific neurobiological processes involved in OFP perception are responsible for a wide range of symptoms that may frequently overlap (1). Clinicians use different classifications for OFP including ICHD-3 (2) although other may underlie clinical decisions, especially for pulpal pain (3). Most of OFP are inflammatory, arising from dental tissues infected by bacteria, which activate intradental neurons. However, the specific innervation of the pulp supports the ability to mimic different pain (4). This article reports a complex case of dental pain presenting as Trigeminal Neuralgia (TN).

## Case Report

A 38 years old male, in general good health, with no history of trauma or medical condition, suddenly experienced severe unilateral rapid-onset pain localized on the maxillary right lateral incisor (tooth #12). At first, he experienced brief episodes of sharp and intense electric shocks-like pain. After 5 days, he was referred by his general practitioner to the dental emergency department of the *Pi*tié Salpêtrière Hospital, where dental students receive and provide care to patients under the supervision of a senior dental dentist.

-1st visit

**Figure 1:**
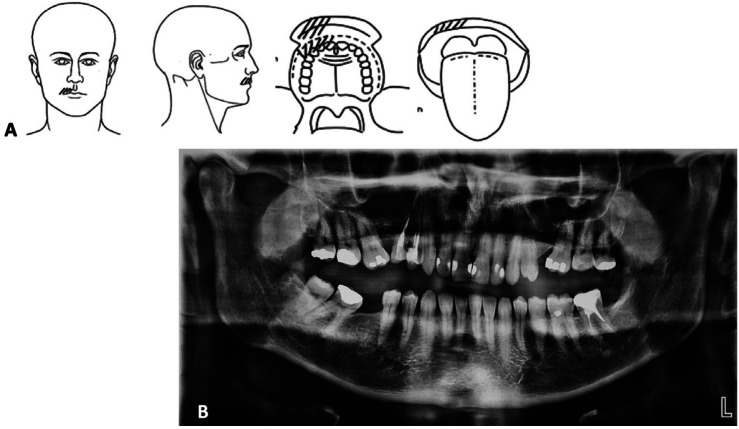
A. Pain drawings. Hatched lines indicated the painful areas described by the patient. B. Panoramic X-Ray. Note the bilateral diastema between teeth #16 and 15 and 24 and 26 with the mesioversion of 24. Teeth 14 and 15 had an endodontic treatment many years ago.

The patient described severe pain attacks, occurring 30 to 50 times/day, lasting less than 3 min, with episodes of total relief in between, located in the anterior right maxilla (Fig. 1A, 2A,B) in the region between the right maxillary central incisor and the first right maxillary premolar, including teeth, bone, gingiva and lip, not attribuTable to a specific tooth. The intensity of pain was rated 9 on a 0-10 numeric scale. Its quality was described mainly as electric shocks and pins and needles. Pain attacks were triggered by innocuous stimuli such as the mere friction of the lip on the tooth during speech.

**Figure 2:**
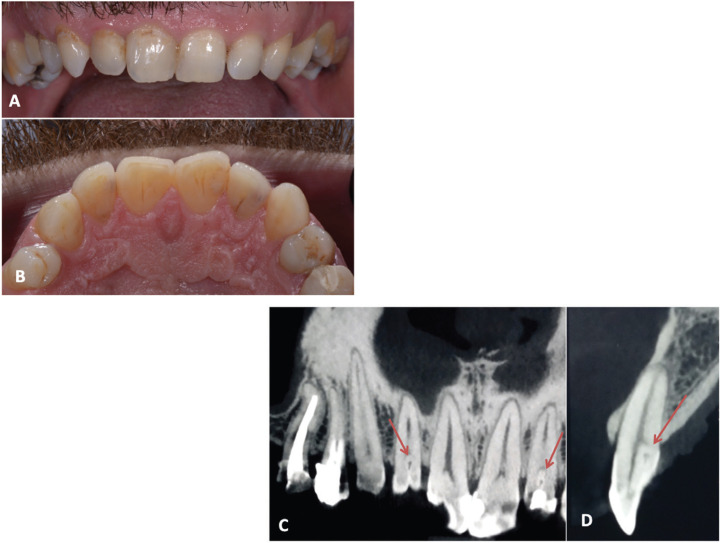
Photographic buccal (A) and palatal (B) view of maxillary teeth. CBCT images (C. frontal view, D. sagittal view) of maxillary teeth. The internal structure is characteristic of a dens invaginatus type II (Ohler’s classification).

Communication and clinical examination were therefore difficult. Thermal, percussion and palpation tests were attempted. Cold test seemed positive for all maxillary teeth. Percussion and palpation were negative although any contact within the area elicited severe pain.

The dental emergency procedure in the GHPS includes a panoramic radiography (OPT) for any doubtful diagnostic conditions. OPT (Fig. 1B) revealed dental absence of teeth #25 and #46, mesioversion of #16, #26 and #47 and a 90º rotation of the tooth 35. The patient reported neither tooth extraction nor orthodontics treatments leading to the conclusion of #25 and #46’s agenesis. No radiolucency was noticed in anterior teeth. Maxillary incisors and molars presented restorative treatments and teeth#14 and #15 were endodontically treated.

According to the pain characteristics fulfilling the ICHD-3 criteria for TN : lasting from a fraction of a second to 2 minutes, with a severe intensity, electric shock-like, shooting, stabbing or sharp in quality, precipitated by innocuous stimuli within the affected trigeminal distribution and not better accounted for by another ICHD-3 diagnosis and the diagnostic tests, a provisional diagnosis of TN affecting the right maxillary branch (V2) of the trigeminal nerve was proposed. Differential diagnoses such as pulpitis, apical periodontitis or other orofacial pain appeared unlikely regarding the responses to clinical tests, the absence of bacterial gateway and the absence of abnormality on the OPT.

A therapeutic and diagnostic medication of carbamazepine 100mg twice a day to increase every 2 days until 600 mg was initiated. A brain MRI was requested in search of vascular nerve compression specific of classical TN or brain invasive processes causing secondary TN. The patient was then referred to a secondary OFP consultation.

-2nd visit

At Day 7, the patient was examined by an OFP specialist (YB). He reported a decrease of his pain 48h after the initiation of the treatment in both intensity and frequency of the crises and was almost painless at D7. The only remaining symptom was a mechanical allodynia affecting tooth #12, i.e. sensitivity to clenching. The dramatic pain relief seemed to corroborate the diagnostic hypothesis of TN; however, the report of the mechanical allodynia was intriguing. Anterior teeth responded positively to the cold test except for tooth #12; whose percussion elicited a non-painful but different sensation compared to the other teeth; electric pulp test response was negative, suggesting pulpal necrosis. Reexamination of panoramic X-Ray elicited doubts related to dental structure, prompting a tomodensitometric examination (CBCT). The carbamazepine treatment was prolonged.

-3rd Visit 

15 days later the patient was still pain free and had stopped his medication. The MRI revealed no abnormality. The maxillary CBCT (Figure 2C-D) evidenced a developmental malformation of teeth #12 and 22 i.e. type II dens invaginatus in Ohler’s classification (5). Pulpal necrosis on tooth #12 was diagnosed and Endodontic Treatment (ET) was advocated.

-4th visit and follow up 

One month after the first consultation, an ET was performed (VM) according to classical procedures. A first follow-up was performed at 2 months, by phone. The patient was pain free, with no mechanical allodynia on tooth #12. At 8 months, X-ray control showed the absence of periapical radiolucency. At one year and 18 months - telephone call follow-ups - the patient was still painless.

## Discussion

This clinical case reports the initial misdiagnosis of a pulpal pain for a trigeminal neuralgia. Both the clinical presentation and the developmental abnormality of the tooth, misguided the clinicians. Several points may explain this confusion and could be of interest for clinicians.

-Symptomatology 

The pain fulfilled the diagnostic criteria for TN in the ICHD3 classification although the patient was not in the usual affected population which is women over 40 years old in 90% of the cases. It was described as electrical shocks, pins and needles and a severe mechanical allodynia was present, which are key features of neuropathic pain. Dental pulpal pain is usually described as throbbing, sharp, dull (3) reflecting inflammatory processes in an enclosed structure. However, neuropathic descriptors, such as shooting, electric-shock, tingling, pins and needles, burning, numbness, are also reported during toothache suggesting that neuropathic mechanisms could contribute to typical toothache pain (6).

The sensory innervation of the dental pulp is mainly composed of small unmyelinated C and myelinated Aδ and of few large diameter Aß fibers and many receptors involved in pain signaling can be found in the dental pulp (7). The specific contribution of dental afferents to sensations is still a matter of debate (8). Stimulation of trigeminal primary afferent nerve fibers by bacteria can lead to the release of numerous bioactive compounds able to alter nerve functioning and trigger phenotypic changes in primary afferents as well as central nervous system (9).

In the reported case, we hypothesize that the pulp was in an irreversible state of inflammation and evolved towards necrosis leading to subsiding of the spontaneous pain after a period of intense pain. This episode was due to the direct stimulation of intradental nerves by the “inflammatory soup” released by inflammation. Although allodynia is a common finding in irreversible pulpitis (10) and can be explained by peripheral mechanisms, such as the sensitization of nerve endings in the periapical tissues surrounding the root end, the severe form reported here might involve central mechanisms i.e. central sensitization and neuroplasticity (9).

An intriguing fact reported here is the paroxysmal, electric discharge-like nature of pain that misguided the clinicians. Paroxysmal pain is associated with damage to Aß fibers (11). Since these are relatively few in the pulp, it might explain why paroxysmal pain is not frequently reported.

-Response to carbamazepine 

Another confusing factor for diagnosis was the decrease of the pain, which seemed due to carbamazepine when it was due to the slow process of necrosis of the pulp. Carbamazepine is the first line drug for TN and its administration is almost considered as a diagnostic test (12). It induces a dramatic decrease of the pain when the patient is responding. The relief of the pain could then be interpreted as an indirect proof of its neuropathic nature. It is also possible that carbamazepine contributed to reduce intradental paroxysmal neuropathic pain trough modulation of action potential generating voltage-gated sodium channels.

-Diagnostic procedures

It is noticeable that the intensity of the pain complicated the differential diagnosis process. Clinical tests, which even in normal conditions can lead to false positive and negative could not be performed properly because even the slightest contact of the tooth was “unbearable”. A local anesthesia test might also provide additional information, although differential diagnosis might be impeded if the anesthesia is performed in the area of the trigger zone. Unfortunately, it would have been the case in this clinical situation. The diagnosis of TN is mainly clinical-based.

-Developmental abnormality

The patient had numerous rare dental characteristics: bilateral dens invaginati in teeth #12 and 22, bilateral premolo-molar diastema, agenesis of 25. This phenotype seems to reflect a developmental genetic anomaly, especially since the patient was being born premature at 6 months although no known syndromic pattern was identified.

## Conclusions

Inflammatory dental pain can mimic pain from other origins such as TN.
